# Quantitative Associations between Season, Month, and Temperature-Humidity Index with Milk Yield, Composition, Somatic Cell Counts, and Microbial Load: A Comprehensive Study across Ten Dairy Farms over an Annual Cycle

**DOI:** 10.3390/ani13203205

**Published:** 2023-10-13

**Authors:** Mostafa Bokharaeian, Abdolhakim Toghdory, Taghi Ghoorchi, Jalil Ghassemi Nejad, Iman Janghorban Esfahani

**Affiliations:** 1 Department of Animal and Poultry Nutrition, Animal Science Faculty, Gorgan University of Agricultural Science and Natural Resources, Gorgan 49189-43464, Iran; mostafa.bokharaeian_s99@gau.ac.ir (M.B.);; 2 Department of Animal Science and Technology, Sanghuh College of Life Sciences, Konkuk University, Seoul 05029, Republic of Korea; 3 Glopex Co., Ltd., R&D Center, GeumGang Penterium IX Tower A2801, Dongtancheomdansaneop 1-ro 27, Hwaseong-si 18469, Gyeonggi-do, Republic of Korea

**Keywords:** heat stress, milk production, temperature–humidity index, somatic cell count, microbial load

## Abstract

**Simple Summary:**

This comprehensive study focused on dairy farms in northeastern Iran to investigate how changing seasons, months, and temperature–humidity index (THI) affect milk production and quality. Data from ten randomly selected dairy herds were collected, including daily milk production records and milk samples for analysis. The study closely examined the influence of season, month, and THI on milk yield, quality, and cow health. Our findings revealed that winter had the highest milk yield, fat, protein, solids-not-fat (SNF), and pH levels, while somatic cell counts (SCC) and total bacterial counts (TBC) were the lowest during this season. The highest values for milk yield, fat, and pH occurred in January, and March showed the highest protein and SNF levels. December had the lowest SCC and TBC values. Our results emphasize the significant impact of THI on milk production and quality, providing valuable insights for effective dairy management, especially in the face of climate change challenges.

**Abstract:**

This current study addresses the knowledge gap regarding the influence of seasons, months, and THI on milk yield, composition, somatic cell counts (SCC), and total bacterial counts (TBC) of dairy farms in northeastern regions of Iran. For this purpose, ten dairy herds were randomly chosen, and daily milk production records were obtained. Milk samples were systematically collected from individual herds upon delivery to the dairy processing facility for subsequent analysis, including fat, protein, solids-not-fat (SNF), pH, SCC, and TBC. The effects of seasons, months, and THI on milk yield, composition, SCC, and TBC were assessed using an analysis of variance. To account for these effects, a mixed-effects model was utilized with a restricted maximum likelihood approach, treating month and THI as fixed factors. Our investigation revealed noteworthy correlations between key milk parameters and seasonal, monthly, and THI variations. Winter showed the highest milk yield, fat, protein, SNF, and pH (*p* < 0.01), whereas both SCC and TBC reached their lowest values in winter (*p* < 0.01). The highest values for milk yield, fat, and pH were recorded in January (*p* < 0.01), while the highest protein and SNF levels were observed in March (*p* < 0.01). December marked the lowest SCC and TBC values (*p* < 0.01). Across the THI spectrum, spanning from −3.6 to 37.7, distinct trends were evident. Quadratic regression models accounted for 34.59%, 21.33%, 4.78%, 20.22%, 1.34%, 15.42%, and 13.16% of the variance in milk yield, fat, protein, SNF, pH, SCC, and TBC, respectively. In conclusion, our findings underscore the significant impact of THI on milk production, composition, SCC, and TBC, offering valuable insights for dairy management strategies. In the face of persistent challenges posed by climate change, these results provide crucial guidance for enhancing production efficiency and upholding milk quality standards.

## 1. Introduction

The dairy industry plays a pivotal role in global agriculture, serving as a primary source of essential nutrition and contributing significantly to economies worldwide [1]. The efficiency and sustainability of dairy production are, however, challenged by a wide range of factors, including climatic changes, which have gained increasing attention due to their profound effects on livestock health and productivity [2]. Among these climatic factors, temperature and humidity levels are key determinants of environmental stressors faced by dairy cattle [3]. As the world experiences shifts in climate patterns and more frequent extreme weather events, understanding the implications of these changes on dairy cattle is of paramount importance.

The temperature–humidity index (THI), a composite measure that takes into account both temperature and humidity, has long been acknowledged as a crucial indicator of thermal stress in livestock. It is derived from air temperature and relative humidity and serves as a comprehensive measure, reflecting the cumulative impact of external factors on the animal’s performance [4,5,6,7,8]. With rising global temperatures and increasing instances of heatwaves, the prevalence of heat stress in dairy cattle is on the rise, necessitating a thorough investigation into its multifaceted impacts on milk quality and udder health.

Changes in milk production and quality, alongside somatic cell counts (SCC) and total bacterial count (TBC), stemming from unfavorable environmental conditions such as heat stress, can yield significant economic consequences for both dairy farmers and processors [9,10,11,12]. Understanding the dynamics of these changes and their underlying mechanisms is essential for developing strategies to maintain milk quality and enhance the overall sustainability of dairy production. Milk quality principally concerns levels of milk fat, protein, and solids-not-fat (SNF). Any undesirable changes in these components result in reduced milk quality [13,14]. Unfavorable environmental conditions are documented to have adverse effects on the body’s metabolism, resulting in reductions in both milk production and quality in lactating animals [15,16,17,18]. These conditions, such as high temperatures, can particularly impact levels of milk fat and protein, making them susceptible to significant fluctuations during seasonal transitions [14,19]. Peana et al. [20] in their previous research have also indicated that elevated temperatures induce variations in milk composition in dairy cows. Somatic cells, as part of the animal’s immune system, play a vital role in safeguarding the mammary glands from infection, making the SCC a key factor in udder health [13,14]. Various factors, including management practices, animal health, milk production levels, stage of lactation, and a range of individual and environmental factors, can influence the milk SCC [21]. In particular, high milk-producing cows, due to the stress of milk production, may experience reduced immunity, resulting in elevated SCC levels in their milk [22]. In a study by M’Hamdi et al. [23], it was observed that the SCC exhibited an increase at high THI levels while decreasing at low THI levels. Additionally, according to Godden et al. [24], elevated heat and humidity levels were found to escalate the pathogen load in the environment, leading to a higher incidence of mastitis and an increased microbial load.

Climate change is suggested to influence milk microbial counts through both direct and indirect mechanisms. Directly, factors such as the THI, which is regarded as the most accurate representation, play an essential role in shifting the milk microbial load [25,26,27]. Moreover, the indirect impact of climate change on milk microbial counts arises from the induction of heat stress in dairy cattle, heightening their vulnerability to pathogenic microbes [28,29].

The current study aims to address the shortage of knowledge concerning the impact of seasons, months, and the THI on milk yield, composition, SCC, and TBC in Iranian dairy farms, mainly in the northeastern regions. Recognizing the significant challenges faced by dairy farmers in maintaining both milk quantity and quality, this research seeks to investigate the effects of environmental factors such as temperature and humidity, encapsulated by THI. Spanning a full annual cycle across ten diverse dairy farms, the hypothesis of the current study was to investigate whether there are intricate relationships between THI and critical parameters such as milk yield, milk fat content, milk protein levels, SNF, SCC, and TBC. This holistic approach promises to provide valuable insights into the complex interactions between environmental conditions, animal health, and milk quality, offering a foundation for more informed and efficient dairy farm management practices.

## 2. Materials and Methods

### 2.1. Experimental Farm Conditions

This study was carried out on ten distinct dairy herds in the northeastern region of Iran (Gonbad-e-Qabus, Golestan, Iran) from January 2021 to January 2022. This region of the country is known for having a Mediterranean climate with hot and humid summers. All the farms included in the study were located within a 50-km radius of Gonbad-e-Qabus (37.25° N, 55.16° E), and as such, their collective data were treated as a single entity. The study encompassed a total of 1566 milking animals, predominantly of the Holstein-Friesian breed, with an average parity of approximately 3. The average number of days in milk (DIM) was 211 days, and the stage of lactation was predominantly in the mid-lactation phase. All the cows were raised under identical management and environmental conditions, residing in an open, spacious barn with a unique design. This barn featured an overshot roof, equipped with a ridge exhaust to facilitate air circulation, as well as fans to promote air exchange during the summer. In the winter months, winch curtains were employed to shield the cows from the chilling effects of the cold wind. Throughout the study, a Total Mixed Ration (TMR) was provided twice daily, at 08:00 a.m. and 04:00 p.m. Efforts were made to maintain the composition of the TMR consistently, but minor adjustments were made based on the farm’s specific needs. The TMR consisted of alfalfa hay, corn silage, wheat straw, a concentrated mixture, barley grain, corn grain, and soybean meal. Additionally, a mineral and vitamin premix were included. Cows had *ad libitum* access to both feed and water, available 24 h a day. On average, the TMR had a composition of 48.6% dry matter (DM), 17.3% crude protein (CP), 4.88% ether extract (EE), 39.2% neutral detergent fiber (NDF), and 19.1% acid detergent fiber (ADF) when measured on a dry matter basis. Additionally, the energy content in the diet for all cows was 1.72 Mcal net energy for lactation per kilogram of DM. Monthly samples of TMR were collected and stored at −20 °C until being analyzed for DM, CP, and EE following the procedures outlined in the AOAC guidelines [30]. Additionally, the NDF and ADF contents were determined using the amylase-treated NDF (aNDF) method as developed by Mertens [31].

### 2.2. Data Collection

A total of ten dairy herds were randomly selected for inclusion in this study. Each herd provided records of their average daily milk production. Additionally, daily milk samples were systematically obtained from the bulk storage tanks of individual herds, precisely upon delivery to the dairy processing facility (Sabah Dairy, Gonbad-e-Qabus, Iran). These collected samples served as the basis for subsequent analytical procedures, including fat content, protein content, SNF, pH, SCC, and TBC. The year was categorized into distinct seasons: spring (from 21 March to 20 June), summer (from 21 June to 20 September), autumn (from 21 September to 20 December), and winter (from 21 December to 20 March) [17]. Records of average daily temperature and relative humidity were obtained from the local weather station in Gonbad-e-Qabus, Iran, located at 37.26° N, 55.20° E. The THI was subsequently calculated using the equation proposed by Marai et al. [32]:

$$THI=T{}^{\circ}C-\left[\left(0.31-0.31RH\right)\left(T{}^{\circ}C-14.4\right)\right]$$

where T°C represents the ambient temperature in degrees Celsius, and RH is relative humidity in percent. As already indicated by Marai et al. [32], the calculated THI values were classified as follows: THI values below 22.2 were considered to indicate the absence of heat stress (AHS), while values ranging from 22.2 to less than 23.3 indicated moderate heat stress (MHS). Furthermore, the THI values between 23.3 and less than 25.6 were demonstrating severe heat stress (SHS), and finally, the THI values equal to or exceeding 25.6 were classified as extreme severe heat stress (ESHS).

### 2.3. Laboratory Analysis

Somatic cell counts were determined using NucleoCounter^®^ SCC-100™ (Allerod, Denmark) and then transformed into the logarithmic [log_2_(SCC × 10^−5^) + 3] somatic cell score (SCS) [33]. The milk samples were promptly tested for TBC. For each sample, eight consecutive dilutions were prepared. The plated surface of each dilution was placed on two plates containing a standard plate count (SPC) medium, which was then cultured for a specific duration. The samples were housed in a controlled greenhouse environment for 72 h at a temperature of 32 °C. Subsequently, the colonies were tallied, and the bacterial count per milliliter of the samples was ascertained [34]. The Milkoscan 134 model (Foss-Electric A/C, Hillerod, Denmark) was employed to assess the milk samples for protein, fat, and SNF in accordance with the IDF (inverse document frequency) Standard 141B:1996. The pH of the milk was measured using the HI981034 Milk pH Tester (HANNA Instruments, Carrollton, TX, USA).

### 2.4. Statistical Analysis

The statistical analysis was performed using the SAS version 9.4 statistical package (SAS Institute Inc., Cary, NC, USA). The effects of seasons, months, and THI on milk yield, composition, SCC, and TBC were assessed using an analysis of variance. Tukey’s test was employed for post-hoc mean separation when statistical significance was detected. To account for these effects, a mixed-effects model was utilized with a restricted maximum likelihood approach, treating month and THI as fixed factors. Pearson correlations were computed between milk yield, various measured milk composition parameters, and environmental factors. The results are reported as least-squares means along with standard deviations, unless explicitly stated otherwise. Statistical significance was determined at a probability level (*p*) of < 0.05, and trends are discussed for variables with *p* ≤ 0.10.

## 3. Results

The summarized statistics for meteorological and production data, including the number, mean, minimum, and maximum of various variables measured during the study, are presented in Table 1.

As presented in Table 2, our investigation revealed noteworthy correlations between key milk parameters and variations in seasons, months, and THI. Positively significant correlations (*p* < 0.01) were observed between milk yield, fat, protein, SNF, and pH with the seasons of the year. In contrast, the correlations between milk SCC and TBC with seasons displayed significant negativity (*p* < 0.01). Aside from SCC (*p* > 0.05), months of the year exhibited significant negative correlations (*p* < 0.01) with milk yield, fat, protein, SNF, pH, and TBC. Significant negative correlations were also observed between THI and milk yield, fat, protein, SNF, and pH, while the correlations between THI with SCC and TBC were significantly positive (*p* < 0.01).

### 3.1. Climatic Conditions

**Figure 1:**
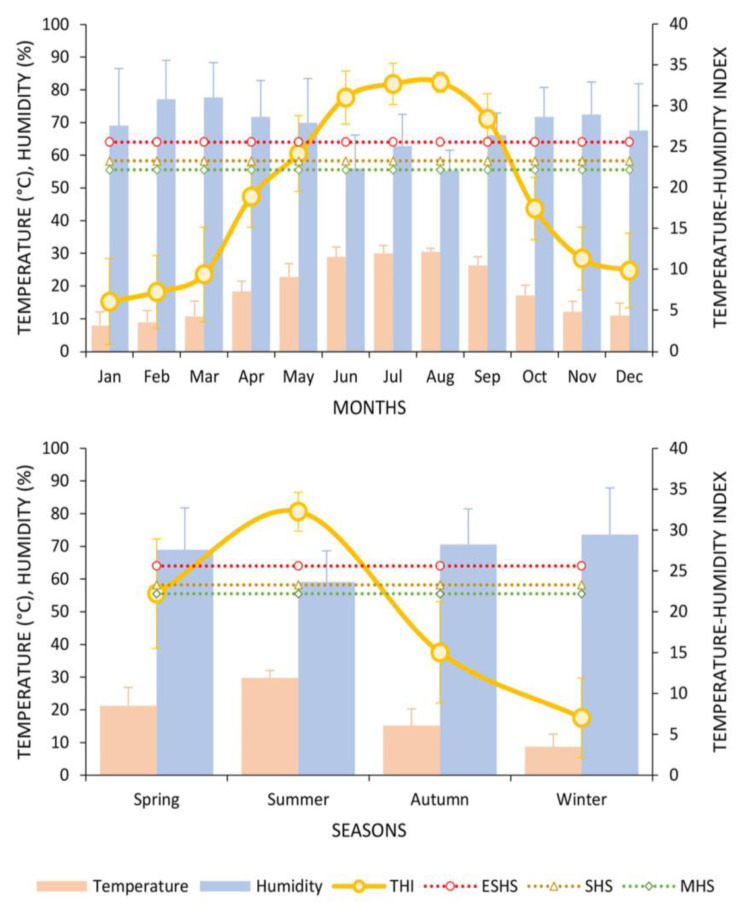
Average values for monthly and seasonal temperature, relative humidity, and THI recorded during 2021.

Figure 1 illustrates the mean values of monthly and seasonal temperature, relative humidity, and the THI recorded throughout the year 2021. During the study period, the mean values for ambient temperature, relative humidity, and THI were recorded as 18.8 °C, 68.0%, and 19.2 units, respectively (Figure 1). The range of monthly THI values was documented, with the minimum and maximum recorded as −3.6 and 37.7 units, respectively (Figure 1). The mean THI reached its minimum value in January (6.1 units) and peaked in August (32.9 units). The average THI exhibited a progression towards severe heat stress in May (spring season), escalating to the category of extremely severe heat stress (ESHS) during the months of June, July, August, and September (summer season).

### 3.2. Effect of Seasons

**Figure 2:**
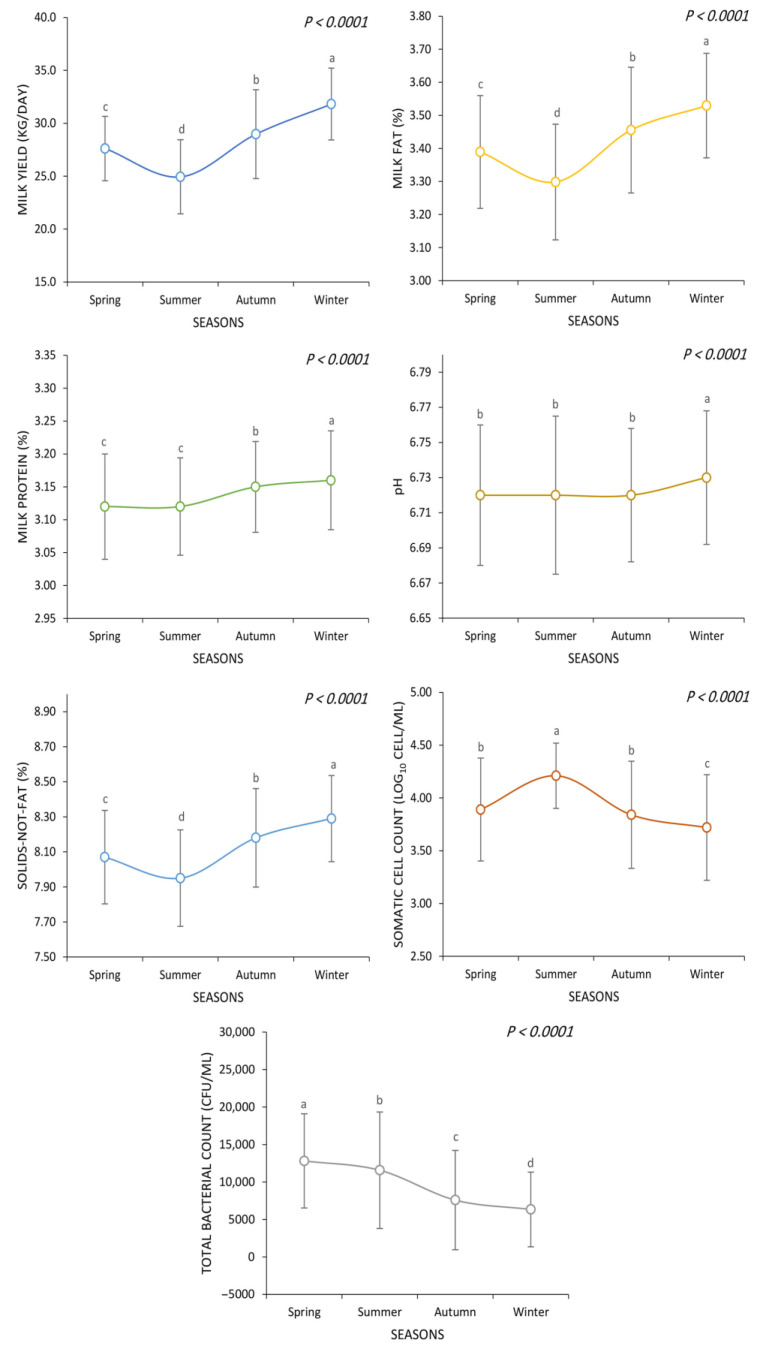
Seasonal variations in milk yield, composition (fat, protein, solids-not-fat), somatic cell count, and total bacterial count. ^a, b, c, d^ Mean values without a common superscript are significantly different (*p* < 0.05).

Figure 2 demonstrates the influence of different seasons on the various experimental parameters, including milk yield, milk composition (fat, protein, SNF), SCC, and TBC. The effect of seasons on various parameters analyzed in the study was statistically significant (*p* < 0.01).

The highest milk yield was observed in winter (31.8 kg/day), followed by autumn (29.0 kg/day), spring (27.6 kg/day), and summer (24.9 kg/day) (*p* < 0.01). In a similar manner, the highest milk fat content was observed in winter (3.53%), followed by autumn (3.46%), spring (3.39%), and summer (3.30%) (*p* < 0.01). In terms of milk protein, the highest value was observed in winter (3.16%), while both spring (3.12%) and summer (3.12%) exhibited the lowest value (*p* < 0.01). Autumn showed an intermediate value (3.15%) for milk protein among the seasons (*p* < 0.01). Milk SNF showed a similar trend as milk yield and milk fat content, with winter demonstrating the highest value (8.29%), followed by autumn (8.18%), spring (8.07%), and summer (7.95%) (*p* < 0.01). There was no significant difference between spring (6.72), summer (6.72), and autumn (6.72) regarding milk pH (*p* > 0.05). However, winter milk had a significantly higher pH value (6.73) (*p* < 0.01). The SCC was the highest in the summer (44.21 log_10_ cell/mL), whereas it was the lowest in the winter (3.72 log_10_ cell/mL) (*p* < 0.01). Both spring (3.89 log_10_ cell/mL) and autumn (3.84 log_10_ cell/mL) showed intermediate values of SCC while they were not significantly different (*p* > 0.05). The highest value of TBC was observed in spring (12,812 CFU/mL), followed by summer (11,574 CFU/mL), autumn (7584 CFU/mL), and winter (6341 CFU/mL) (*p* < 0.01).

### 3.3. Effect of Months

**Figure 3:**
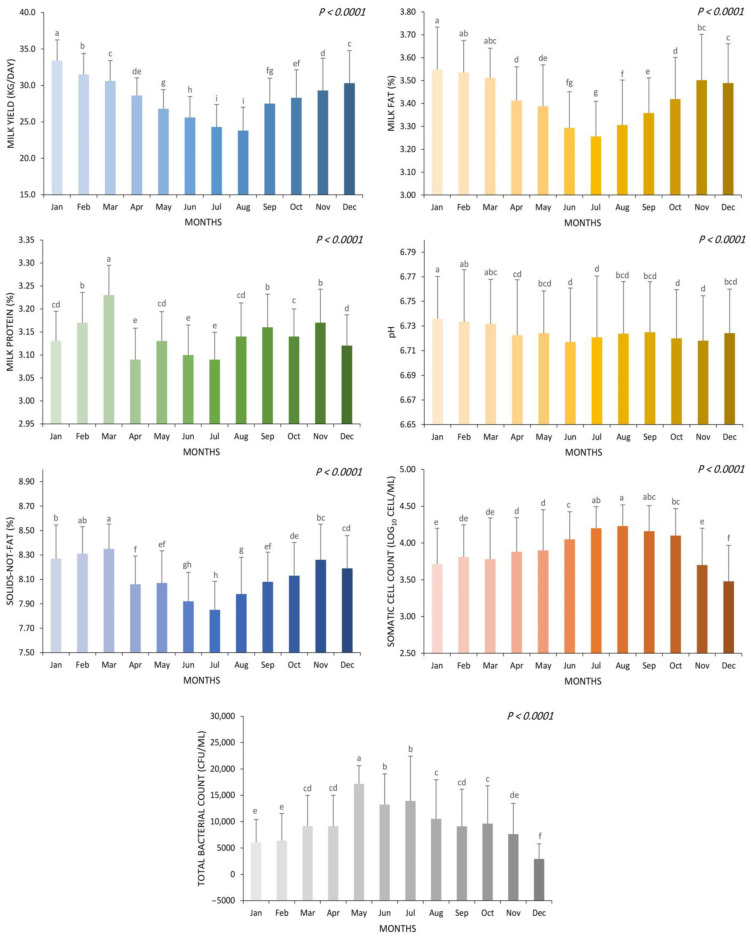
The effect of months on the average milk yield, composition (fat, protein, solids-not-fat), somatic cell count, and total bacterial count. ^a, b, c, d, e, f, g, h, i^ Mean values without a common superscript are significantly different (*p* < 0.05).

Figure 3 illustrates the pronounced impact of varying months on multiple aspects of the investigation, encompassing milk yield, composition (fat, protein, solids-not-fat), milk SSC, and TBC. Every parameter analyzed in the study exhibited statistically significant differences across the various months of the year (*p* < 0.01). Remarkably, the highest milk yield was recorded during January (33.4 kg/day), followed closely by February (31.5 kg/day), whereas the lowest milk yield was concurrently recorded in both July (24.3 kg/day) and August (23.8 kg/day) (*p* < 0.01). There was also a notable trend in milk fat content across the months studied. The highest fat content was observed in January (3.55%), closely followed by February (3.54%) and March (3.51%). In contrast, the lowest fat content was recorded in July (3.26%) (*p* < 0.01). The protein content exhibited significant temporal variation, with the highest value observed in March (3.23%), and the lowest values occurring in April (3.09%), June (3.10%), and July (3.09%) (*p* < 0.01). In terms of SNF content, the highest value was observed in March (8.35%), closely followed by February (8.31%) and January (8.27%). In contrast, the lowest SNF content was recorded in July (7.85%), with a significant difference (*p* < 0.01). The results reveal a distinct trend in milk pH levels across the months. Notably, the highest milk pH value was observed in January (6.74), whereas the lowest levels were consistently recorded in June, July, October, and November, each registering a pH of 6.72 (*p* < 0.01). The SCC exhibited significant temporal variability, with the highest count observed in August (4.23 log_10_ cells/mL), contrasting sharply with the lowest count registered in December (3.48 log_10_ cells/mL) (*p* < 0.01). The TBC exhibited a clear seasonal pattern, with the highest levels recorded in May (17,161 CFU/mL), followed by June (13,248 CFU/mL) and July (13,922 CFU/mL). Notably, the lowest TBC was observed in December (2912 CFU/mL) (*p* < 0.01), revealing a significant fluctuation in microbial abundance throughout the year.

### 3.4. Effect of THI

**Figure 4:**
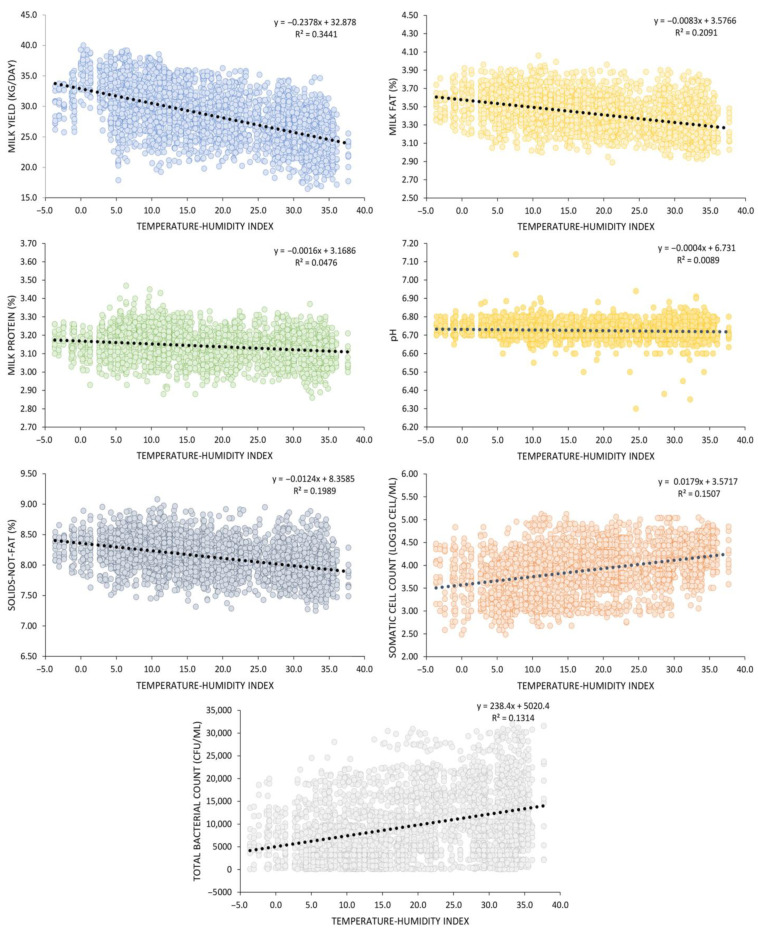
The effect of THI on the average milk yield, composition (fat, protein, solids-not-fat), somatic cell count, and total bacterial count.

Figure 4 illustrates the relationship between THI and various milk parameters, including milk yield, milk fat, milk protein, SNF, SCC, and TBC, while Table 2 complements this by reporting the corresponding correlation coefficients. Across the THI spectrum, spanning from −3.6 to 37.7, distinct trends were evident. A linear regression model, y = −0.2378x + 32.878, explained 34.41% of the variance in milk yield due to THI, revealing a strong inverse correlation (r = −0.59, *p* < 0.01). Similarly, a linear model captured the variation in milk fat content, with y = −0.0083x + 3.5766, and an R^2^ of 0.2091. Notably, a significant negative correlation was observed between THI and milk fat (r = −0.46, *p* < 0.01). For milk protein, a linear regression model, y = −0.0016x + 3.1686, yielded an R^2^ of 0.0476 and a weak negative correlation (r = −0.22, *p* < 0.01). Analogously, a linear model, y = −0.0124x + 8.3585, with an R^2^ of 0.1989, described the relationship between THI and SNF, which displayed a significant negative correlation (r = −0.45, *p* < 0.01). Moreover, the interaction between THI and pH was examined through a linear regression, y = −0.0004x + 6.7310, with an R^2^ of 0.0089, revealing a significant negative correlation (r = −0.10, *p* < 0.01). Notably, SCC exhibited an upward trend with increasing THI, as evidenced by the linear regression y = 0.0179x + 3.5717, demonstrating an R^2^ of 0.1507 and a significant positive correlation (r = 0.39, *p* < 0.01). Concerning TBC, a linear model, y = 238.4x + 5020.4, fits the data moderately (R^2^ = 0.1314), suggesting a linear relationship with THI. Furthermore, the correlation coefficient between THI and TBC was 0.36 (*p* < 0.01), denoting a moderately positive association.

## 4. Discussion

The comprehensive investigation of milk yield and composition in association with seasons, months, and THI offers valuable insights into the dynamic correlation between environmental factors and dairy outcomes. Our findings, which revealed significant correlations among these variables, shed light on the multifaceted nature of dairy farming in tropical regions. The seasonal variations observed in milk yield and composition underscore the influence of changing climatic conditions on dairy herd performance. Lactating animals are considered highly susceptible to elevated temperatures and humidity levels. The production of milk in these animals and their milk yield are closely associated with shifts in climate patterns, marked by prolonged exposure to substantial heat and humidity spanning approximately 6 months each year [35]. The THI serves as a comprehensive indicator for evaluating the degree of heat and cold stress in dairy cows, representing the combined influence of temperature and relative humidity.

### 4.1. Seasonal Trends in Milk Yield and Quality

Our study reveals distinct seasonal patterns in milk production. Notably, the highest milk yield was observed during the winter, while the lowest occurred in the summer. These findings are consistent with previous research. Yoon et al. [36] reported a 0.93 kg/day higher milk yield during the winter compared to the summer. Similarly, Bernabucci et al. [37] reported a 0.50 kg/day higher milk yield during the winter as opposed to the summer. The strong positive correlation of 0.43 between season and milk yield signifies a moderate linear relationship, implying that milk yield tends to rise with changing seasons. However, the moderate correlation strength implies the involvement of additional influencing factors. Consistent with our findings, Bernabucci et al. [37] and Bertocchi et al. [38] observed elevated levels of fat, protein, and SNF in milk during the winter, contrasting with decreased concentrations during the summer. As proposed by Bertocchi et al. [38], a potential explanation for the decrease in fat and protein content during the warmer seasons (spring and summer) is the elevated occurrence of calving and, consequently, a greater population of recently calved cows in comparison to other seasons. Lim et al. [39] similarly documented a progressive rise in milk protein levels among Holstein cows from spring to winter. In their investigation, Lim et al. [39] demonstrated a 6.2% reduction in average milk yield among Holstein cows as the average THI values transitioned from spring to autumn, potentially due to heightened energy expenditure. The intensified heat production might contribute to the accelerating decline in milk yield. Furthermore, the extent of the permanent decrease in ongoing lactation is directly linked to the duration of heat stress. Furthermore, variations in milk production across seasons arise from cyclic environmental changes throughout the year. Bohmanova et al. [4] describe these variations as arising from both direct impacts on milk production, characterized by reduced dry matter intake, and indirect effects stemming from fluctuations in feed availability and quality.

Our observations indicate elevated SCC during the summer, aligning with the findings of Lim et al. [39], who found a significant increase in SCC during the summer in Holstein cows. Negative correlations between seasons and SCC, as well as TBC, underscore the potential challenges associated with specific climatic conditions, which could impact both udder health and milk quality. Alhussien and Dang [21] similarly noted a mild seasonal effect on milk SCC, with higher values observed in the summer compared to the winter and spring seasons.

### 4.2. Monthly Trends in Milk Yield and Quality

Months played an essential role in shaping the trends we observed in milk production and its composition, notably fat, SNF, and, to some extent, protein levels. Our findings revealed that these key indicators reached their highest values during the colder months of the year. These results were further supported by significant negative correlations linking milk yield and its constituents with temperature, as well as significant negative correlations with the month. This was in line with the findings of Zhu et al. [35], who reported an increase in daily milk production in January, February, and March, contrasting with the lowest daily yields in July and August. These results underscore the significance of temperature in influencing the daily milk yield of ruminants. In the study conducted by Md. Akkas et al. [40], a notable trend emerged wherein milk yield exhibited an increasing pattern during the months coinciding with decreasing THI values. This was confirmed by the significant negative correlation we observed between the month and milk yield. Yang et al. [41] documented a remarkably similar monthly pattern in milk fat, protein, and milk solids, mirroring the observations from our current investigation. Nonetheless, minor disparities could potentially be attributed to variations in environmental and geographical conditions across the two experiments. Consistency in these findings was further supported by the work of Barash et al. [42], where cows demonstrated their highest daily milk yield in February and the lowest in September. The most substantial reduction in milk yield was observed between June and September (summer), while a rebound was evident from October to November (autumn), indicative of a recovery phase. Our previous study also highlighted elevated milk fat content during January, February, and March [43], in line with Kljajevic et al. [44], underscoring the profound influence of environmental temperature on milk composition. Our results corroborate this observation, as we identified stronger correlation coefficients between milk fat and both month and temperature compared to other constituents.

### 4.3. Effect of Temperature-Humidity Index

Temperature–humidity index profoundly influences milk production and various aspects of milk composition and SCC, as evidenced by several studies. Md. Akkas et al. [40] revealed a noteworthy trend where increased THI levels were associated with a statistically significant decrease in milk production, milk fat, and milk SNF. Notably, they identified an inverse relationship between milk fat and SNF levels with respect to THI levels. Lower THI values consistently correlated with higher milk fat and SNF, in contrast to instances of higher THI. These observations corroborated our own research outcomes, further substantiated by a notable and statistically significant negative correlation established between THI and both milk fat and SNF levels. Lee et al. [45] observed a reduction in each milk component when the THI surpassed the specific threshold. Furthermore, the literature suggests that milk fat tends to decrease in response to heat stress (HS) conditions [46,47,48]. Bouraoui et al. [49] proposed that this decrease might be linked to reduced forage consumption in the diet. They argued that TMR could mitigate heat stress-induced milk fat depression by maintaining the balance between forage and concentrate intake and ensuring sufficient fiber for proper rumen function. Moallem et al. [50] pointed out that the primary adverse effect of elevated THI levels is the reduction in rumination time, subsequently leading to decreased dry matter intake and, consequently, reduced milk yield. The release of somatic cells in milk is influenced by various factors, including milk productivity, animal health, management practices, and environmental conditions, as elaborated upon in the following discussion [21]. High-milk-producing cows experience increased stress due to production demands, compromising their immunity and resulting in elevated SCC in their milk [22]. This effect is especially pronounced in high-producing cows during hot and humid seasons compared to their mid- and low-producing counterparts, indicating increased udder stress during this period [51]. Lee et al. [45] reported a significant increase in SCC in cows when the THI exceeded the breakpoint, a finding mirrored by Hagiya et al. [52], who observed a marked increase in SCC beyond the THI threshold. These investigations are consistent with the results of our own study. Micronutrient deficiencies arising from poor-quality fodder can contribute to the heightened growth of infectious bacteria, coupled with diminished immunity and elevated SCC [21].

## 5. Conclusions

In conclusion, our findings highlight the paramount influence of the THI on milk production, constituents, SCC, and TBC. We observed a significant negative correlation between THI and milk constituents (fat, protein, and SNF levels), providing practical insights for dairy management practices. The implications extend to udder health, as high-producing cows, especially during hot and humid seasons, exhibit heightened stress, compromising immunity and resulting in elevated SCC. This relationship between THI and SCC reinforces the importance of proactive herd management to mitigate SCC risks during environmental stress periods. As the dairy industry confronts ongoing challenges linked to climate change, these findings offer essential guidance for optimizing production efficiency and maintaining milk quality standards. Further research in this field promises to refine our knowledge, enhancing the sustainability of dairy operations in a dynamically changing environment.

## Figures and Tables

**Table 1:** Descriptive statistics of milk composition, microbial load, and somatic cell count in bulk milk tanks of 10 herds in 2021.

Variable	Label	N	Mean	Std Dev	Minimum	Maximum
Temperature, °C	T°C	3650	18.8	8.94	0.5	35.1
Relative Humidity, %	RH	3650	68.0	13.19	31.0	97.4
Temperature-Humidity Index	THI	3650	19.2	10.69	−3.6	37.7
Milk Yield, kg/day	MY	3650	28.3	4.33	16.5	40.0
Milk Fat, %	MF	3650	3.42	0.194	2.89	4.06
Milk Protein, %	MP	3650	3.14	0.077	2.86	3.47
Solids-Not-Fat, %	SNF	3650	8.12	0.297	7.25	9.08
pH	pH	3650	6.72	0.041	6.30	7.14
Somatic Cell Count, log_10_ cells/mL	SCC	3650	3.92	0.493	2.49	5.12
Total Bacterial Count, CFU/mL	TBC	3650	9601	7031.8	100	31,970

**Table 2:** Analysis of correlation coefficients between measured parameters of milk yield, milk composition and environmental factors.

	Items	1	2	3	4	5	6	7	8	9	10	11	12	13
1	Herd	**1.00**												
2	Seasons	0.00	**1.00**											
3	Months	0.00	−0.05 **	**1.00**										
4	AT	0.00	−0.65 **	0.23 **	**1.00**									
5	RH	0.00	0.21 **	−0.15 **	−0.59 **	**1.00**								
6	THI	0.00	−0.65 **	0.23 **	1.00 **	−0.58 **	**1.00**							
7	MY	−0.60 **	0.43 **	−0.24 **	−0.59 **	0.28 **	−0.59 **	**1.00**						
8	Fat	0.09 **	0.33 **	−0.13 **	−0.46 **	0.22 **	−0.46 **	0.25 **	**1.00**					
9	Protein	−0.17 **	0.21 **	−0.05 **	−0.22 **	0.15 **	−0.22 **	0.23 **	0.43 **	**1.00**				
10	SNF	0.02 ^ns^	0.34 **	−0.12 **	−0.44 **	0.23 **	−0.45 **	0.28 **	0.96 **	0.66 **	**1.00**			
11	pH	−0.11 **	0.10 **	−0.10 **	−0.09 **	0.05 **	−0.10 **	0.13 **	0.03 ^ns^	0.02 ^ns^	0.03 *	**1.00**		
12	SCC	−0.27 **	−0.20 **	0.03 ^ns^	0.39 **	−0.19 **	0.39 **	−0.08 **	−0.17 **	0.01 ^ns^	−0.13 **	0.01 ^ns^	**1.00**	
13	TBC	0.17 **	−0.37 **	−0.07 **	0.36 **	−0.11 **	0.36 **	−0.32 **	−0.23 **	−0.11 **	−0.22 **	−0.04 *	0.13 **	**1.00**

AT, ambient temperature; RH, relative humidity; THI, temperature-humidity index; MY, milk yield; SNF, solids-not-fat; SCC, somatic cell count; TBC, total bacterial count. *, **, and ^ns^ indicate *p* < 0.05, *p* < 0.01, and non-significant (*p* > 0.05), respectively.

## Data Availability

Data are available upon a reasonable request.
